# A clinical metagenomic study of biopsies from Mexican endophthalmitis patients reveals the presence of complex bacterial communities and a diversity of resistance genes

**DOI:** 10.1099/acmi.0.000639.v3

**Published:** 2024-06-20

**Authors:** Miguel Ángel Vences-Guzmán, Martín Jiménez-Rodríguez, Luis Lozano, Sergio Rojas-Juárez, Juan Abel Ramírez-Estudillo, Ángeles Yahel Hernández-Vázquez, Ingrid Yazmín Pita-Ortiz, Karol Guadalupe Ramírez-Ceballos, Silvia Medina-Medina, Christian Sohlenkamp

**Affiliations:** 1 Centro de Ciencias Genómicas, Universidad Nacional Autónoma de México, Cuernavaca, Morelos, Mexico; 2 Departamento de Retina, Fundación Hospital Nuestra Señora de la Luz IAP, Mexico City, Mexico

**Keywords:** endophthalmitis, moxifloxacin, resistome, shotgun metagenomics

## Abstract

Infectious endophthalmitis is a severe ophthalmic emergency. This infection can be caused by bacteria and fungi. For efficient treatment, the administration of antimicrobial drugs to which the microbes are susceptible is essential. The aim of this study was to identify micro-organisms in biopsies of Mexican endophthalmitis patients using metagenomic next-generation sequencing and determine which antibiotic resistance genes were present in the biopsy samples. In this prospective case study, 19 endophthalmitis patients were recruited. Samples of vitreous or aqueous humour were extracted for DNA extraction for metagenomic next-generation sequencing. Analysis of the sequencing results revealed the presence of a wide variety of bacteria in the biopsies. Resistome analysis showed that homologues of antibiotic resistance genes were present in several biopsy samples. Genes possibly conferring resistance to ceftazidime and vancomycin were detected in addition to various genes encoding efflux pumps. Our findings contrast with the widespread opinion that only one or a few bacterial strains are present in the infected tissues of endophthalmitis patients. These diverse communities might host many of the resistance genes that were detected, which can further complicate the infections.

**Impact Statement**

Metagenomic next-generation sequencing reveals the presence of complex microbial communities in biopsies of endophthalmitis patients, which contrasts with the widely held belief that these infections are caused by single strains. Genes possibly conferring resistance to ceftazidime and vancomycin were detected in addition to a variety of genes encoding efflux pumps, and the presence of these resistance genes can further complicate the infections.

## Data Summary

The authors confirm that all supporting data, code and protocols have been provided within the article or through supplementary data files. The sequencing data were deposited at NCBI with the SRA accession number: SRP424451. Bioproject: PRJNA937315PRJNA937315. Biosamples: SAMN33401590, SAMN33401589, SAMN33401588, SAMN33401587, SAMN33401586, SAMN33401585, SAMN33401584, SAMN33401583, SAMN33401582.

## Introduction

Infectious endophthalmitis is a severe ophthalmic emergency. It is a rare but serious complication of cataract surgery with an incidence of about 0.1 %, frequently leading to poor visual outcomes [1,4]. This infection can be caused by bacteria and fungi, and for efficient treatment, the administration of antimicrobial drugs to which the microbes are susceptible is essential. Since the publication of the Endophthalmitis Vitrectomy Study (EVS), the current recommendations for empirical therapy have not changed [5]. Many of the common causative organisms are sensitive to the broad-spectrum antibiotic combination vancomycin and ceftazidime, which is the current standard, but there are reports of cases of infectious endophthalmitis resistant to these antibiotics. According to the EVS, only 89.5 % of Gram-negative isolates are sensitive to amikacin and ceftazidime. Furthermore, the increased prevalence of methicillin-resistant bacteria has become a clinical problem. New antibiotics that can be administered intravitreally are required, and other types of antibiotics could be used to control intraocular infections [6]. One alternative antibiotic that could be used in endophthalmitis patients is the fourth-generation fluoroquinolone moxifloxacin (MXF). MXF has been tested in *in vitro* studies using cell culture and in animal models. Previous studies have established that 150 µg ml^−1^ MXF is a safe concentration for rabbit eyes and *in vitro* human pigmented cells. Intravitreal MXF caused no electroretinographic or retinal histological abnormalities [7,8].

The recognition of the causative pathogens of acute endophthalmitis is fundamental and can have wide-reaching implications because a more specific treatment can be selected [9]. Traditional culture- and microscope-based methods remain the gold standard, but the vitreous tap volume is limited, and traditional culture-based methods need a relatively large sample volume to grow bacteria [10]. Most pathogens that were cultured from biopsies are Gram-positive, such as *Staphylococcus aureus*, *Staphylococcus epidermis* and *Streptococcus viridans*, but some Gram-negative isolates such as *Pseudomonas aeruginosa* have also been grown from biopsies [10]. Using these classical methods, if successful, it takes several days to determine the infectious agent, but an additional complication is that a large proportion of the micro-organisms cannot be cultured [9,11]. In earlier studies when culture-based methods for pathogen identification were used, often only a single pathogen was identified, and these results seemed to imply that infectious endophthalmitis is caused by a single bacterium or fungus. Using PCR-based methods for pathogen identification, only pathogens that will be looked for can be identified, so the general idea of a single pathogen being responsible did not change [12,14]. More recently, detection methods based on next-generation sequencing (NGS) have allowed for a more detailed and hypothesis-free analysis of the micro-organisms present in a sample. One of two general strategies is followed: targeted amplicon sequencing, for example of the 16S rRNA gene, or microbial whole-genome shotgun sequencing [15,16]. Deep sequencing of fragments of the 16S rRNA gene was used with success to detect unculturable bacteria and to reduce the time of detection of pathogens [15]. Pathogens were detected in several samples from which it had not been possible to culture bacteria. A disadvantage is that the detection of a specific 16 rRNA only shows if a certain genus or species is present, but it does not reveal which resistance genes are present in the microbial population. To tackle this problem, shotgun metagenomic approaches are the best way to predict to which antibiotics a certain population is resistant and which treatment to choose [17,20]. To gain a comprehensive understanding of the different types, mechanisms, transmission and evolution of antibiotic resistance one needs to understand the contribution of the whole microbiota. While our study was in progress, Zhu *et al*. [21] published a metagenomic NGS study to detect pathogens in endophthalmitis patients. They detected thousands of species of micro-organisms, making it difficult to identify the causal agent, but despite these results they claimed to have identified one or few causal pathogens per sample. It was concluded that NGS was more efficient than culture-based methods to identify pathogens, but they also noted that the NGS results were not always concordant with culture-based approaches [21].

Here, we present a metagenomic DNA shotgun sequencing study of biopsies obtained from Mexican endophthalmitis patients to describe the micro-organisms present and to identify the causal agents. After the sample was obtained patients were treated with a combination of ceftazidime/vancomycin or with MXF. Total DNA was purified from biopsies and sequenced. The resulting sequencing dataset was analysed by assembly of the short reads into larger contiguous DNA fragments and by mapping the sequencing reads to reference sequences. This method allowed the determination of the microbiota composition and was used to detect the antibiotic resistance genes present.

## Methods

### Clinical study and methods

This prospective case series study was approved by the internal review board of the Fundación Hospital Nuestra Señora de la Luz IAP, Mexico City. It was conducted in accordance with the Declaration of Helsinki and the purpose of the Good Clinical Practice Guidelines. All patients were informed of the experimental nature of the intervention, including a detailed description of the ‘Gold standard therapy’ with vancomycin and ceftazidime, possible complications of the disease, and the pros and cons of the experimental therapy. All patients signed an informed consent form before participating in the study.

We included male and female patients over 18 years of age, regardless of the cause [post-surgery, corneal ulcer, intravitreal injection (IVI), open globe trauma], with a clinical diagnosis of acute endophthalmitis. We excluded patients with no light perception visual acuity or with tissue lysis that required evisceration. Demographic data, laterality, type of surgery and days of symptom presentation were recorded.

After enrolment, all patients underwent a full ophthalmological examination which included an assessment of initial best-corrected visual acuity (BCVA), measured in logMAR units, slit lamp examination [conjunctival hyperemia, corneal epithelial damage, intraocular pressure, lens status, anterior chamber cells, anterior chamber haze and vitreous haze (using SUN Grading scheme for each one) [22]. If the anterior chamber or vitreous were not clear for clinical examination, an A/B scan ultrasound examination was realized. Pain as a variable was measured with an analog visual scale score.

In the operating room, vitreous tap was obtained, and 0.1 ml MXF was injected via pars plana into the vitreous under topical anaesthesia with draping in a routine sterile fashion. Samples were immediately refrigerated at 4 °C, then mixed with glycerol to a final concentration of 20 % (w/v) and stored in a deep freezer.

Patients underwent IVI of 0.1 ml MXF every 48 h if required until the ophthalmological examination showed signs of improvement (cornea haze reduction, anterior chamber inflammation decreased). Pars plana vitrectomy 25G with the Stellaris PC Platform (Bausch and Lomb) was then realized under retrobulbar anaesthesia. All patients were draped in a sterile fashion. The eyelid skin was disinfected with 10 % povidone iodine (PI) and the conjunctiva was disinfected with 4 % PI. Silicon oil was used as vitreous tamponade at the end of the surgery. Sclerotomies were sutured with 7–0 polyglactin (Vicryl; Ethicon). Moxifloxacin drops and prednisolone acetate were used after the surgery.

Patients were followed daily after IVI with MXF, variables of clinical examination were compared day 1 to the day previous to the vitrectomy in each case and final BCVA was determined 1 month after the surgery. Doses of MXF, time to the vitrectomy and follow-up time were recorded in each file.

### Preparation of MXF solution for IVI

In this study we used commercial sealed MXF solution (Vigamoxi 0.5 %; Alcon; 500 µg/0.1 ml) for IVI. This formula was reported to be preservative-free and has a low toxic concentration for intraocular use in previous *in vitro* reports and case reports. A 0.1 ml aliquot of MXF (500 µg/0.1 ml) in the average vitreous volume of 4 ml corresponds to an empiric concentration of 125 µg ml^−1^. It has a half-life time of 1.72 h [7,8]. The MIC90 of fluoroquinolones in one case report was low, ≤1 µg ml^−1^ for ciprofloxacin and ≤2 µg ml^−1^ for levofloxacin, which might explain the clinical resolution of infections despite the short half-life time of this class of antibiotic [23,24].

### Samples and DNA extraction

Genomic DNA was isolated from 100 µl of vitreous or aqueous fluid using the Qiagen blood and tissue DNA extraction kit according to the manual. When less than 100 µl sample volume was available, the total volume was used for DNA extraction. The DNA was eluted in the kit elution buffer and stored at −20 °C. DNA concentration was determined using a NanoDrop ND-1 000 spectrophotometer (Thermo Scientific).

### Sequencing, metagenome assembly and taxonomic classification

DNA was sequenced at INMEGEN (Nacional Institute of Genomic Medicine, Mexico City; samples 2, 3, 6, 7, 8) or at Omega Bioservices (Norcross, GA, USA; samples 15, 16, 17, 19). Paired-end sequencing was performed on MiSeq (250 bp ×2) or HiseqX10 (150 bp ×2) platforms. Quality analysis of the Illumina sequencing files was performed with FastQC (RRID:SCR_014583, https://www.bioinformatics.babraham.ac.uk/projects/fastqc/) software version 0.11.9; low-quality bases and adapters were removed with Trim Galore (RRID:SCR_011847, https://www.bioinformatics.babraham.ac.uk/projects/trim_galore/) software version 0.6.6, and the first 15 bases of the reads of all fast files were removed because or erroneous base sequence content. The human genome sequence version 38 was obtained from the refseq ftp site of the NCBI [25], this genome was indexed with Bowtie2build and the Illumina reads were aligned with Bowtie2 [26] software v2.3.4.1. (RRID:SCR_016368, http://bowtie-bio.sourceforge.net/bowtie2/index.shtml). The Illumina reads that aligned concordantly to the human genome were excluded from the fastq files to obtain the human filtered fastq files.

*De novo* genome assembly was performed for each metagenome sample with SPAdes [27] software version 3.14.1 with metagenome option and kmer values of 21, 33, 55, 77, 99, 111 and 127, using the human filtered fastq files. The assembled contigs were used for gene prediction and annotation with Prokka [28] version 1.12 with the metagenome option. Taxonomic classification was performed with Kraken2 [25] software version 2.0.8-beta using the assembled contigs with the mini-kraken2_v1_8 Gb database with the -use-mpa-style option. The Kraken2 results were analysed and visualized with Pavian [29] software version 1.2.0. The protein sequences of all metagenome samples were blasted [30] against the last version of the full dataset Virulence Factor Database (VFDB) [31] and the protein homologue model type of the Comprehensive Antibiotic Resistance Database (CARD) [32]. Blast hits with these two databases were considered significant if the protein query and hit have 80 % alignment coverage and 40 % sequence identity.

## Results and discussion

### Clinical information

From June 2018 to August 2019, 19 patients with endophthalmitis were evaluated. Their mean age was 63.6±16.2 years; four patients (21.0 %) were female and 15 patients (78.9 %) were male. In nine patients (47.3 %) the left eye was affected. Two patients had an open wound, and 17 cases were associated with ophthalmological procedures. In nine eyes cataract surgery was the potential cause of infection, in four eyes IVI, in two eyes corneal ulcer, in one eye penetrating keratoplasty and in one eye vitrectomy was the cause. The mean time from surgery to the start of symptoms was 7.2±8.4 days. Four patients were treated with a combination of ceftazidime and vancomycin and the other 15 with MXF. The mean initial visual acuity was 2.48 logMAR (hands motion or 20/4 000), and at the end, the mean VA was 1.8 logMAR (count fingers or 20/1 000). However, we registered patients with a 20/20 visual acuity at the end. Only one patient showed a worsened clinical status, but for the others, intravitreal antibiotics improved corneal oedema enough to realize an early pars plana vitrectomy. Demographic and clinical information of the included cases is given in Table 1. The total number of sequenced samples was low, but as this post-surgical complication is not very frequent, samples are very difficult to obtain and frequently are of low volume. In addition, to our knowledge this is only the second study of this type of sample and the first with samples from Latin America.

**Table 1.** Summary of clinical data M, male; F, female. R, right; L, left. NLP, no light perception; LP, light perception; HM, hands movement; CF, count fingers. VA, visual acuity. IVI, intravitreal injection.

No.	Sex	Eye	Age (years)	Aetiologies	Initial VA	Time of symptoms(days)	Final VA	IVI (No.)	Antibiotic	Complementarytreatment	Sample	Most abundant bacteria identified by mNGS
1	M	R	44	Penetrating keratoplasty	HM	6	NLP	2	Ceftazidime+vancomicin	Evisceration	Vitreous	Not sequenced
META2	M	R	79	Corneal ulcer	LP	1	LP	6	Ceftazidime+vancomicin	None	Vitreous	***Pasteurella multocida***
META3	M	L	71	Intravitreal injection	LP	7	HM	2	Moxifloxacin	Vitrectomy	Vitreous	***Staphylococcus aureus***
4	F	R	63	Cataract surgery	LP	30	20/20	2	Ceftazidime+vancomicin	Vitrectomy	Vitreous	Not sequenced
5	M	L	48	Vitrectomy	LP	3	LP	1	Moxifloxacin	Vitrectomy	Vitreous	Not sequenced
META6	M	R	38	Open wound	HM	6	20/400	2	Moxifloxacin	Wound closure	Vitreous	***Streptococcus pneumoniae***
META7	M	R	51	Cataract surgery	LP	30	20/70	2	Ceftazidime+vancomicin	Vitrectomy	Vitreous	***Pasteurella multocida***
META8	M	L	87	Cataract surgery	20/40	4	20/30	1	Moxifloxacin	None	Vitreous	***Pandoera norimbergensis***
9	M	L	83	Intravitreal injection	CF	4	20/200	2	Moxifloxacin	Phacovitrectomy	Vitreous	Not sequenced
10	M	L	35	Corneal ulcer	LP	3	CF	2	Moxifloxacin	None	Vitreous	Not sequenced
11	M	R	82	Cataract surgery	LP	3	CF	1	Moxifloxacin	Vitrectomy	Vitreous	Not sequenced
12	M	R	70	Intravitreal injection	20/80	1	20/80	1	Moxifloxacin	None	Vitreous	Not sequenced
13	F	L	81	Cataract surgery	LP	3	HM	2	Moxifloxacin	Vitrectomy	Vitreous	Not sequenced
14	M	R	68	Cataract surgery	HM	7	20/200	2	Moxifloxacin	Vitrectomy	Vitreous	Not sequenced
C15	F	L	69	Intravitreal injection	LP	10	HM	2	Moxifloxacin	Phacovitrectomy	Vitreous	***Sphingomonas* sp**.
C16	F	R	68	Cataract surgery	LP	1	NLP	1	Moxifloxacin	None	Vitreous	***Klebsiella pneumoniae***
C17	M	L	65	Cataract surgery	CF	6	20/50	1	Moxifloxacin	Vitrectomy	Vitreous	***Klebsiella pneumoniae***
18	M	L	65	Cataract surgery	CF	6	20/50	1	Moxifloxacin	Vitrectomy	Aqueous	Not sequenced
C19	M	R	42	Open wound	LP	6	HM	2	Moxifloxacin	Phacovitrectomy	Vitreous	***Klebsiella pneumoniae***

### Effect of moxifloxacin treatment

After the full ophthalmological exam, a vitreous tap sample was obtained, and antibiotic treatment was initiated. Three of the first patients to present as candidates for the protocol accepted vitreous sample collection but not the application of MXF; the remaining patients accepted both interventions. As seen in Table 1, one of the patients treated with the dual antibiotic had to undergo evisceration due to poor progression. Within the MXF group, all patients showed clinical improvement that allowed advancement to a pars plana vitrectomy, enabling a complementary surgical approach for resolution. We conclude that the use of MXF, compared to the use of dual antibiotics, was not inferior, did not worsen the condition, and allowed for surgical intervention to improve the structural and functional conditions of the patient. Therefore, MXF can be considered an additional alternative for treating this highly disabling pathology, aggressive to ocular tissue, and requiring diverse diagnostic and management alternatives due to its presentation with multiple pathological agents, as observed in this study. A detailed description of the cases is beyond the scope of this publication and will be published in a separate article.

### DNA extraction and sequencing

A fundamental challenge is that only small volumes of intraocular fluid can be safely obtained, and, in our study, this sometimes was not enough to extract sufficient DNA for successful library construction and sequencing. DNA extraction for metagenomic shotgun sequencing can be difficult in samples with lower microbial biomass and high human DNA content [33]. Some samples contain human tissue fragments, whereas others do not. Some samples may contain high numbers of bacterial cells, whereas others do not. This means that sample characteristics can differ from sample to sample, which could explain the different amounts of DNA that could be extracted from the biopsies. In one case, no DNA could be detected after extraction of the sample and from other samples only minimum amounts not sufficient for library construction were obtained. In the nine samples that could be sequenced we obtained between 2.2 and 191 million sequencing reads (Table 2). Sequencing was done at two different sequencing facilities, which is the main reason for the difference in sequencing depth reported. The first five samples were sequenced at a Mexican non-commercial institution, and the final four samples were sent abroad. As expected, a large fraction of the reads were assigned to human DNA. Depending on the sample between 16.6 and 62.4 % of the sequencing reads were therefore excluded from further analysis. Previous studies had observed between 81 and 99 % human reads [34]. From these filtered reads between 12 000 and 1.8 million contigs could be assembled, but only between 2.4 and 38 % of the assembled contigs could be classified properly by Kraken2 (Table 2).

**Table 2.** General information about the sequencing results Total reads: number of reads obtained from Illumina sequencing; Filtered reads: number of reads that remained after total reads were aligned versus human genome v38; Contigs: number of contigs assembled with the filtered reads; Assembly: megabases assembled; Predicted genes: number of genes predicted from the assembled contigs; Classified contigs: number of contigs properly classified with Kraken2.

	Total reads	Filtered reads	Contigs	Assembly (Mb)	Predicted genes	Classified contigs
META2	12 556 288	9 918 778	107 336	37	13 105	4 157
META3	16 001 022	13 349 320	128 614	44	16 150	5 749
META6	7 131 962	5 390 718	58 883	22	8 768	2 933
META7	2 282 582	1 585 174	84 756	30	12 422	4 806
META80	18 880 270	8 929 374	12 944	3	2 150	4 949
C15	143 536 508	54 014 296	1 714 050	583	108 786	45 872
C16	191 525 902	76 121 928	1 371 027	1 323	321 403	86 959
C17	135 897 938	52 257 342	1 834 288	592	111 796	45 122
C19	116 364 188	45 113 668	1 265 077	389	72 102	32 133

### Metagenome sequencing shows that a complex mixture of bacteria and virus can be detected in the biopsies

**Fig. 1.**
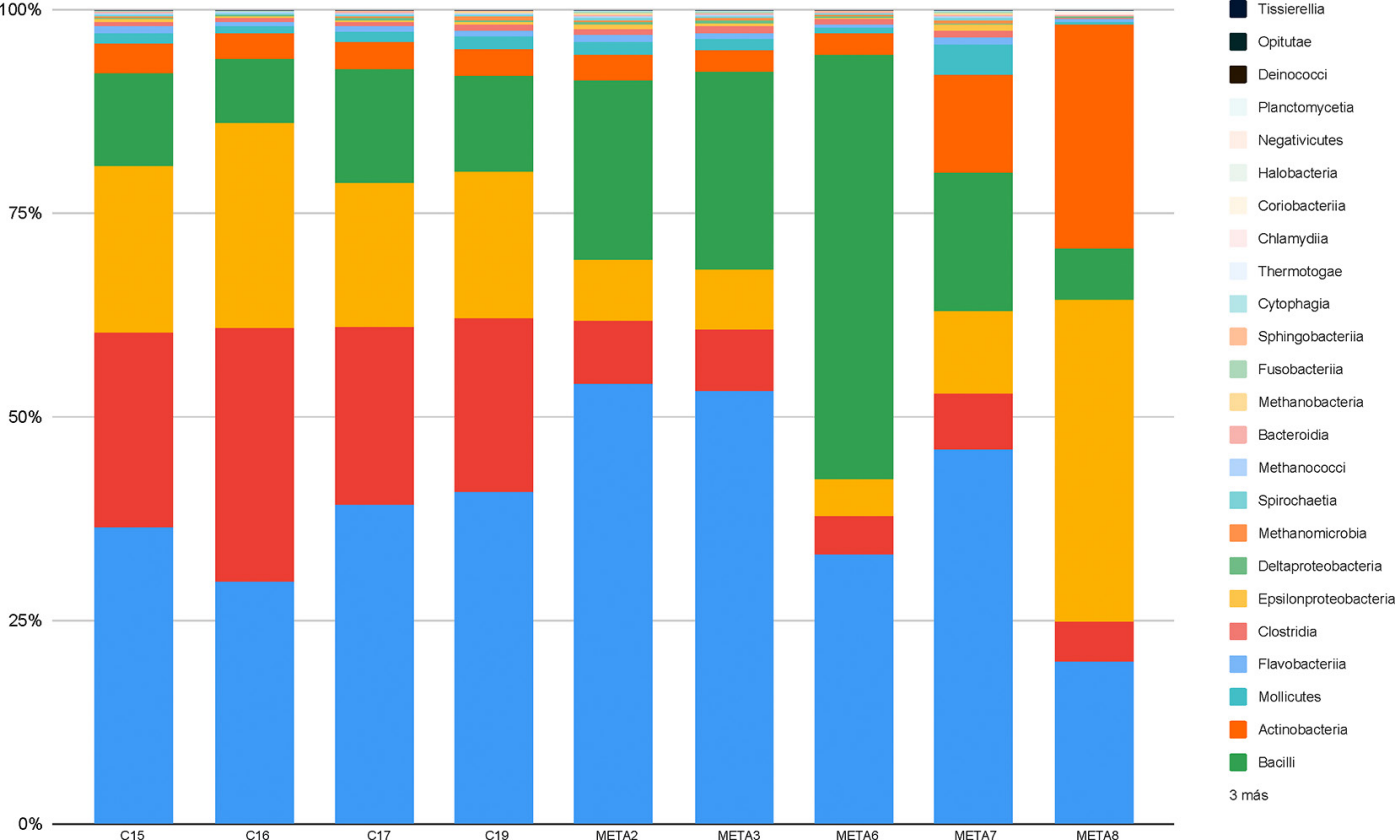
Relative abundance of sequencing reads assigned to taxonomic classes. Samples from left to right: META2, META3, META6, META7, META8, C15, C16, C17 and C19.

**Fig. 2.**
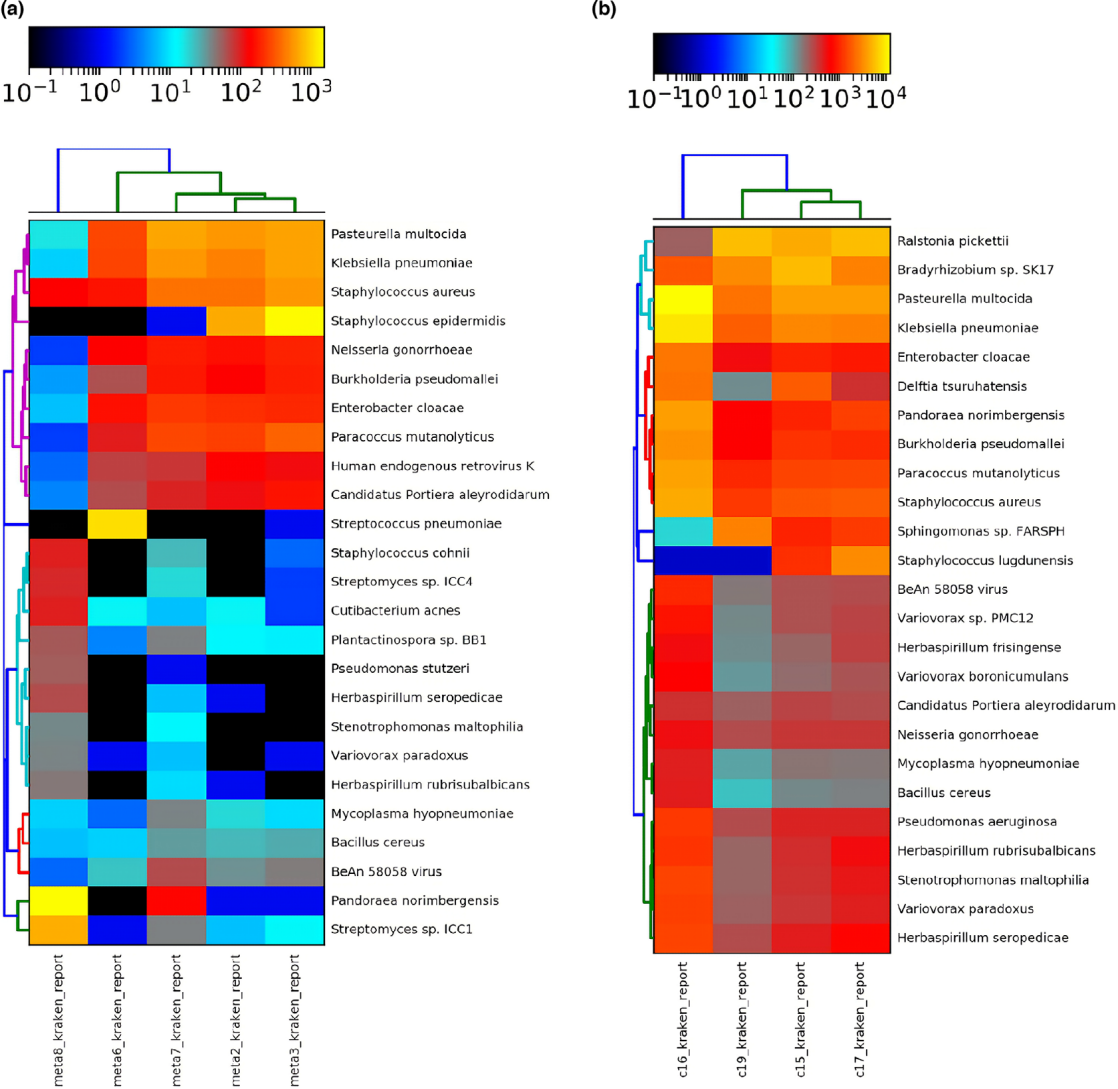
Heatmaps showing the 25 most abundant micro-organisms and virus per sample. (a) Samples META2, 3, 6, 7 and 8. (b) Samples C15, 16, 17 and 19. The top bar in both panels represents the mapping of the numerical values to a colour scale.

After assembling the sequencing reads into contigs, these were assigned to taxonomic classes. By abundance, six major taxonomic classes were detected in each of the biopsies: *Betaproteobacteria*, *Gammaproteobacteria*, *Alphaproteobacteria*, *Bacilli*, *Actinobacteria* and *Mollicutes*. In addition, smaller numbers of reads and contigs could be assigned to other taxonomic classes. The relative amounts of the taxonomic classes varied substantially (Fig. 1) but already at this point in our analysis we noted that the sample compositions were complex and by no means were monocultures dominated by a single bacterium. In previous studies mainly Gram-positive bacteria were isolated from endophthalmitis patients, but in the samples from Mexican patients that we analysed, most bacteria detected were proteobacteria (Fig. 1). We then tried to assign the contigs at the species level and it was confirmed that a large diversity of bacteria was present in the biopsies (Table 3 and Fig. 2). Many of the bacteria detected in this study are known as pathogens or opportunistic pathogens and are identical to typical bacteria identified by earlier studies using culture-dependent approaches or PCR-based approaches as causal agents of the infection. *Staphylococcus aureus*, *Staphylococcus epidermidis*, *Streptococcus pneumoniae*, *Pandoraea norimbergensis*, *Ralstonia pickettii*, *Pasteurella multocida*, *Neisseria gonorrhoe*, *Burkholderia pseudomallei*, *Enterobacter cloacae*, *Klebsiella pneumoniae*, *Paracoccus mutanolyticus, Streptomyces* sp. ICC1, *Bradyrhizobium* sp. SK17, *Delftia tsuruhatensis, Sphingomonas* sp. FARSPH, *Stenotrophomonas maltophilia*, *Herbaspirillum seropedicae, Variovorax paradoxus* and *Pseudomonas aeruginosa* were among the bacterial species most frequently detected across the samples (Fig. 2 and Table 3). Additionally, some of the bacterial genera we detected are known pathogens but have been also described to be part of the core genera at the ocular surface such as *Pseudomonas*, *Staphylococcus*, *Streptococcus* and *Sphingomonas*. Other genera that we detected, such as *Ralstonia* and *Delftia*, are known to contain pathogenic strains causing infection in other tissues [35]. We were surprised to detect *Bradyrhizobium* sp. SK17 in many of our samples, but this genus, best known for members that are nodule-forming bacteria on the roots of legume plants [36], has also been described as a typical component of the core microbiome of the ocular surface [35]. In our study, in eight out of nine samples more than half of the detected bacteria were proteobacteria, which is high compared to previous studies. In European and North American reports, in 4–6 % of the cases Gram-negative bacteria were the cause of endophthalmitis, but in Asian, Chinese and Indian studies this number increased to over 20 % [37,43]. The normal microbiota of the healthy ocular surface and the pathogens of an infected eye vary widely, depending on the age and immune condition of the patient, geographical location and ambient climate [34].

**Table 3.** List of microbial species identified (the ten best hits of each of the nine samples, including number of contigs assigned, total reads assigned to these contigs and taxonomic class to which the species belong)

**Organism**	**Contigs**	**Reads**	**Taxonomy (Phylum/Class/Order/Family)**
**C15**			
*Bradyrhizobium* sp. SK17	5 624	30 320	Proteobacteria/Alphaproteobacteria/Rhizobiales/Bradyrhizobiaceae
*Ralstonia pickettii*	4 724	71 370	Proteobacteria/Betaproteobacteria/Burkholderiales/Burkholderiaceae
*Pasteurella multocida*	4 030	102 394	Proteobacteria/Gammaproteobacteria/Pasteurellales/Pasteurellaceae
*Klebsiella pneumoniae*	3 068	108 983	Proteobacteria/Gammaproteobacteria/Enterobacterales/Enterobacteriaceae
*Delftia tsuruhatensis*	1 864	11 959	Proteobacteria/Betaproteobacteria/Burkholderiales/Comamonadaceae
*Staphylococcus aureus*	1 782	80 197	Firmicutes/Bacilli/Bacillales/Staphylococcaceae
*Paracoccus mutanolyticus*	1 414	37 479	Proteobacteria/Alphaproteobacteria/Rhodobacterales/Rhodobacteraceae
*Burkholderia pseudomallei*	1 151	31 727	Proteobacteria/Betaproteobacteria/Burkholderiales/Burkholderiaceae
*Staphylococcus lugdunensis*	1 120	8 093	Firmicutes/Bacilli/Bacillales/Staphylococcaceae
*Sphingomonas* sp. FARSPH	994	113 763	Proteobacteria/Alphaproteobacteria/Sphingomonadales/Sphingomonadaceae
**C16**			
*Pasteurella multocida*	12 412	144 821	Proteobacteria/Gammaproteobacteria/Pasteurellales/Pasteurellaceae
*Klebsiella pneumoniae*	9 085	155 777	Proteobacteria/Gammaproteobacteria/Enterobacterales/Enterobacteriaceae
*Staphylococcus aureus*	4 531	6 723	Firmicutes/Bacilli/Bacillales/Staphylococcaceae
*Paracoccus mutanolyticus*	4 325	51 030	Proteobacteria/Alphaproteobacteria/Rhodobacterales/Rhodobacteraceae
*Pandoraea norimbergensis*	4 038	24 963	Proteobacteria/Betaproteobacteria/Burkholderiales/Burkholderiaceae
*Burkholderia pseudomallei*	3 518	42 284	Proteobacteria/Betaproteobacteria/Burkholderiales/Burkholderiaceae
*Enterobacter cloacae*	2 502	50 080	Proteobacteria/Gammaproteobacteria/Enterobacterales/Enterobacteriaceae
*Delftia tsuruhatensis*	2 443	16 603	Proteobacteria/Betaproteobacteria/Burkholderiales/Comamonadaceae
*Bradyrhizobium* sp. SK17	1 779	70 608	Proteobacteria/Alphaproteobacteria/Rhizobiales/Bradyrhizobiaceae
*Herbaspirillum seropedicae*	1 430	6 723	Proteobacteria/Betaproteobacteria/Burkholderiales/Oxalobacteraceae
**C17**			
*Ralstonia pickettii*	5 869	43 215	Proteobacteria/Betaproteobacteria/Burkholderiales/Burkholderiaceae
*Pasteurella multocida*	4 026	98 841	Proteobacteria/Gammaproteobacteria/Pasteurellales/Pasteurellaceae
*Staphylococcus lugdunensis*	3 230	22 24	Firmicutes/Bacilli/Bacillales/Staphylococcaceae
*Klebsiella pneumoniae*	2 939	102 706	Proteobacteria/Gammaproteobacteria/Enterobacterales/Enterobacteriaceae
*Bradyrhizobium* sp. SK17	2 875	13 542	Proteobacteria/Alphaproteobacteria/Rhizobiales/Bradyrhizobiaceae
*Staphylococcus aureus*	1 848	76 732	Firmicutes/Bacilli/Bacillales/Staphylococcaceae
*Paracoccus mutanolyticus*	1 462	34 541	Proteobacteria/Alphaproteobacteria/Rhodobacterales/Rhodobacteraceae
*Pandoraea norimbergensis*	1 340	11 481	Proteobacteria/Betaproteobacteria/Burkholderiales/Burkholderiaceae
*Sphingomonas* sp. FARSPH	1 270	103 007	Proteobacteria/Alphaproteobacteria/Sphingomonadales/Sphingomonadaceae
*Burkholderia pseudomallei*	1068	29225	Proteobacteria/Betaproteobacteria/Burkholderiales/Burkholderiaceae
**C19**			
*Ralstonia pickettii*	5 887	46 946	Proteobacteria/Betaproteobacteria/Burkholderiales/Burkholderiaceae
*Bradyrhizobium* sp. SK17	3 256	14 164	Proteobacteria/Alphaproteobacteria/Rhizobiales/Bradyrhizobiaceae
*Sphingomonas* sp. FARSPH	2 947	75 473	Proteobacteria/Alphaproteobacteria/Sphingomonadales/Sphingomonadaceae
*Pasteurella multocida*	2 491	83 872	Proteobacteria/Gammaproteobacteria/Pasteurellales/Pasteurellaceae
*Klebsiella pneumoniae*	1 900	88 916	Proteobacteria/Gammaproteobacteria/Enterobacterales/Enterobacteriaceae
*Staphylococcus aureus*	1 219	65 525	Firmicutes/Bacilli/Bacillales/Staphylococcaceae
*Paracoccus mutanolyticus*	1 009	30 730	Proteobacteria/Alphaproteobacteria/Rhodobacterales/Rhodobacteraceae
*Pandoraea norimbergensis*	688	7 040	Proteobacteria/Betaproteobacteria/Burkholderiales/Burkholderiaceae
*Burkholderia pseudomallei*	655	25 737	Proteobacteria/Betaproteobacteria/Burkholderiales/Burkholderiaceae
*Enterobacter cloacae*	555	32 366	Proteobacteria/Gammaproteobacteria/Enterobacterales/Enterobacteriaceae
**META2**			
*Staphylococcus epidermidis*	704	5 054	Firmicutes/Bacilli/Bacillales/Staphylococcaceae
*Pasteurella multocida*	574	23 897	Proteobacteria/Gammaproteobacteria/Pasteurellales/Pasteurellaceae
*Klebsiella pneumoniae*	474	22 816	Proteobacteria/Gammaproteobacteria/Enterobacterales/Enterobacteriaceae
*Staphylococcus aureus*	411	22 920	Firmicutes/Bacilli/Bacillales/Staphylococcaceae
*Paracoccus mutanolyticus*	247	9 692	Proteobacteria/Alphaproteobacteria/Rhodobacterales/Rhodobacteraceae
*Enterobacter cloacae*	220	10 142	Proteobacteria/Gammaproteobacteria/Enterobacterales/Enterobacteriaceae
*Neisseria gonorrhoeae*	161	1 943	Proteobacteria/Betaproteobacteria/Neisseriales/Neisseriaceae
Human endogenous retrovirus K	144	2 048	Viruses/Ortervirales/Retroviridae
*Burkholderia pseudomallei*	137	7 926	Proteobacteria/Betaproteobacteria/Burkholderiales/Burkholderiaceae
Candidatus Portiera aleyrodidarum	120	8 580	Proteobacteria/Gammaproteobacteria/Oceanospirillales/Halomonadaceae
**META3**			
*Staphylococcus epidermidis*	1 472	10 039	Firmicutes/Bacilli/Bacillales/Staphylococcaceae
*Pasteurella multocida*	655	31 951	Proteobacteria/Gammaproteobacteria/Pasteurellales/Pasteurellaceae
*Klebsiella pneumoniae*	650	31 442	Proteobacteria/Gammaproteobacteria/Enterobacterales/Enterobacteriaceae
*Staphylococcus aureus*	573	32 303	Firmicutes/Bacilli/Bacillales/Staphylococcaceae
*Paracoccus mutanolyticus*	354	13 693	Proteobacteria/Alphaproteobacteria/Rhodobacterales/Rhodobacteraceae
*Enterobacter cloacae*	203	13 356	Proteobacteria/Gammaproteobacteria/Enterobacterales/Enterobacteriaceae
*Neisseria gonorrhoeae*	193	2 459	Proteobacteria/Betaproteobacteria/Neisseriales/Neisseriaceae
*Burkholderia pseudomallei*	185	11 100	Proteobacteria/Betaproteobacteria/Burkholderiales/Burkholderiaceae
Candidatus Portiera aleyrodidarum	163	11 983	Proteobacteria/Gammaproteobacteria/Oceanospirillales/Halomonadaceae
Human endogenous retrovirus K	121	2 698	Viruses/Ortervirales/Retroviridae
**META6**			
*Streptococcus pneumoniae*	1 155	42 636	Firmicutes/Bacilli/Lactobacillales/Streptococcaceae
*Pasteurella multocida*	266	12 824	Proteobacteria/Gammaproteobacteria/Pasteurellales/Pasteurellaceae
*Klebsiella pneumoniae*	263	12 239	Proteobacteria/Gammaproteobacteria/Enterobacterales/Enterobacteriaceae
*Staphylococcus aureus*	163	10 520	Firmicutes/Bacilli/Bacillales/Staphylococcaceae
*Enterobacter cloacae*	158	5 653	Proteobacteria/Gammaproteobacteria/Enterobacterales/Enterobacteriaceae
*Neisseria gonorrhoeae*	135	1 011	Proteobacteria/Betaproteobacteria/Neisseriales/Neisseriaceae
*Paracoccus mutanolyticus*	104	5 165	Proteobacteria/Alphaproteobacteria/Rhodobacterales/Rhodobacteraceae
Human endogenous retrovirus K	74	1 139	Viruses/Ortervirales/Retroviridae
Candidatus Portiera aleyrodidarum	67	2 752	Proteobacteria/Gammaproteobacteria/Oceanospirillales/Halomonadaceae
*Burkholderia pseudomallei*	65	4 255	Proteobacteria/Betaproteobacteria/Burkholderiales/Burkholderiaceae
**META7**			
*Pasteurella multocida*	667	11 656	Proteobacteria/Gammaproteobacteria/Pasteurellales/Pasteurellaceae
*Klebsiella pneumoniae*	566	9 186	Proteobacteria/Gammaproteobacteria/Enterobacterales/Enterobacteriaceae
*Staphylococcus aureus*	416	9 613	Firmicutes/Bacilli/Bacillales/Staphylococcaceae
*Paracoccus mutanolyticus*	272	4 103	Proteobacteria/Alphaproteobacteria/Rhodobacterales/Rhodobacteraceae
*Enterobacter cloacae*	235	3 174	Proteobacteria/Gammaproteobacteria/Enterobacterales/Enterobacteriaceae
*Neisseria gonorrhoeae*	181	760	Proteobacteria/Betaproteobacteria/Neisseriales/Neisseriaceae
*Burkholderia pseudomallei*	171	2 912	Proteobacteria/Betaproteobacteria/Burkholderiales/Burkholderiaceae
*Pandoraea norimbergensis*	149	2 038	Proteobacteria/Betaproteobacteria/Burkholderiales/Burkholderiaceae
Candidatus Portiera aleyrodidarum	99	3 834	Proteobacteria/Gammaproteobacteria/Oceanospirillales/Halomonadaceae
Human endogenous retrovirus K	84	670	Viruses/Ortervirales/Retroviridae
**META8**			
*Pandoraea norimbergensis*	1 566	88 901	Proteobacteria/Betaproteobacteria/Burkholderiales/Burkholderiaceae
*Streptomyces* sp. ICC1	735	27 546	Actinobacteria/Actinobacteria/Streptomycetales/Streptomycetaceae
*Staphylococcus aureus*	138	5 270	Firmicutes/Bacilli/Bacillales/Staphylococcaceae
*Staphylococcus cohnii*	103	5 415	Firmicutes/Bacilli/Bacillales/Staphylococcaceae
*Cutibacterium acnes*	102	32 414	Actinobacteria/Actinobacteria/Propionibacteriales/Propionibacteriaceae
*Streptomyces* sp. ICC4	94	3 583	Actinobacteria/Actinobacteria/Streptomycetales/Streptomycetaceae
*Herbaspirillum seropedicae*	68	3 383	Proteobacteria/Betaproteobacteria/Burkholderiales/Oxalobacteraceae
*Plantactinospora* sp. BB1	60	5 296	ctinobacteria/Actinobacteria/Micromonosporales/Micromonosporaceae
*Pseudomonas stutzeri*	58	4 605	Proteobacteria/Gammaproteobacteria/Pseudomonadales/Pseudomonadaceae
*Herbaspirillum rubrisubalbicans*	44	2 694	Proteobacteria/Betaproteobacteria/Burkholderiales/Oxalobacteraceae

As the torque teno virus was identified in a few patients with culture-negative endophthalmitis [44], we were also interested in the viral reads we could detect. We looked specifically for reads that could be mapped to virus. In some samples we detected BeAn 58 058 virus, which belongs to the *Poxviridiae*. It was first isolated in 1963 from the blood of a rodent of the genus *Oryzomis* in a tropical rain forest, in the region of Belém-do-Pará, Brazil [45]. Interestingly, this virus was also detected in the lung microbiome from patients with chronic obstructive pulmonary disease [46]. Other viral reads were detected in much lower numbers or were only present in very few samples.

We also looked specifically for reads and contigs that could be mapped to fungi. *Malassezia restricta* was detected in five samples (C15=7 contigs, C16=13 contigs, C17=4 contigs, C19=6 contigs, META6=2 contigs). In a few samples single contigs of *Fusarium graminearum* (19), *Pyricularia pennisetigena* (8 and 16), *Neurospora crassa* (17) and *Sugiyamaella lignohabitans* (16) were detected. Deshmukh *et al*. had also observed fungi from the genus *Malassezia* in several samples of endophthalmitis patients [47] and *Fusarium* sp. had also been described in previous studies [48].

The results from the assignment of the sequencing contigs to bacterial species were used to prepare two different heat maps to further characterize and compare the different samples. The division was made because it allowed an easier interpretation despite the large differences in sequencing reads between the two groups of samples (Fig. 2). Sequencing depth has a major impact on the results of metagenomic shotgun sequencing [49]. At decreased sequencing depth, the number of undetected microbial species increases together with unclassified and misclassified clades. The large bacterial diversity that we and others [10] detected in the biopsies complicates the identification of the causal agent for the infection. It is important to emphasize that our data showed dramatically different prevalence and much greater diversity at the genus level than typically revealed by classical culture-based methods (Table 3 and Fig. 2a and b). At least for our small number of samples, it is not a single bacterial species that is dominant, but we observed complex microbiomes.

In our shotgun sequencing study, many bacterial species were identified in all nine samples that could be sequenced. This is an observation usually completely missed by culture-based methods [47]. Often, the bacteria involved appear to originate from the patients’ own microbiota but may also be introduced through contaminated solutions or instruments used during ophthalmological procedures [50]. Pathogenic bacteria identified in other studies as causal agents were identified but were often not among the most abundant micro-organisms. A complication of this observation is that it is unclear which bacteria are mainly responsible for the infection and the tissue damage. Other bacterial species normally not known to be involved in pathogenesis might, however, influence the process of infection because they might harbour antibiotic resistance genes that could be transferred horizontally to the pathogens.

The heterogeneity of the biopsies did not allow successful extraction of DNA in all cases. In some cases, enough DNA could be extracted, but in others not. Clearly, this is a problem when suggesting metagenomic shotgun sequencing for diagnosis. Also, metagenomic shotgun sequencing is currently still too time-consuming and methodologically too complex to become a routine method. We think that recent methodological developments such as Nanopore sequencing may have a future application for diagnostic purposes that can improve the quality of care of patients with infectious endophthalmitis. This type of sequencing allows results to be obtained in a shorter timeframe. Recently, an article was published describing a turnover time from 7 to 9 h from sample collection until an informed decision for sepsis treatment using Nanopore sequencing [51].

### Vancomycin resistance genes and a large diversity of efflux pumps are detected by metagenome sequencing

Antibiotic resistance is spreading worldwide, and this situation is especially worrying in clinical settings. For the efficient treatment of patients with endophthalmitis, the identification of the causal agent(s) is important in choosing the correct treatment. Most common antibiotics target conserved mechanisms of bacterial metabolism such as cell wall synthesis (beta-lactams, vancomycin), protein synthesis (tetracycline and neomycin), DNA replication (fluoroquinolones) and single carbon metabolism such as folate synthesis (sulfonamides) [19]. Bacteria become resistant by four major mechanisms: enzymatic degradation or modification of the antibiotic, alteration of the antibiotic target, active efflux of the antibiotic and reduced permeability of the bacterial cell surface. The resistance mechanisms can be specific or they can be more general; for example, a beta-lactamase will only act on beta-lactam antibiotics, whereas an efflux pump can export different antibiotics out of the cell or a cell wall modification affecting the cell wall permeability can make a cell less permeable for several antibiotics [19]. Metagenomic deep sequencing allows us to identify specific antibiotic resistance genes present in a microbial population [18,52, 53]. Frequently, these resistance genes are located on mobile genetic elements, and this allows rapid spread of the resistance in a bacterial population [54], but which can complicate the assignment of a resistance gene to a specific species.

**Fig. 3.**
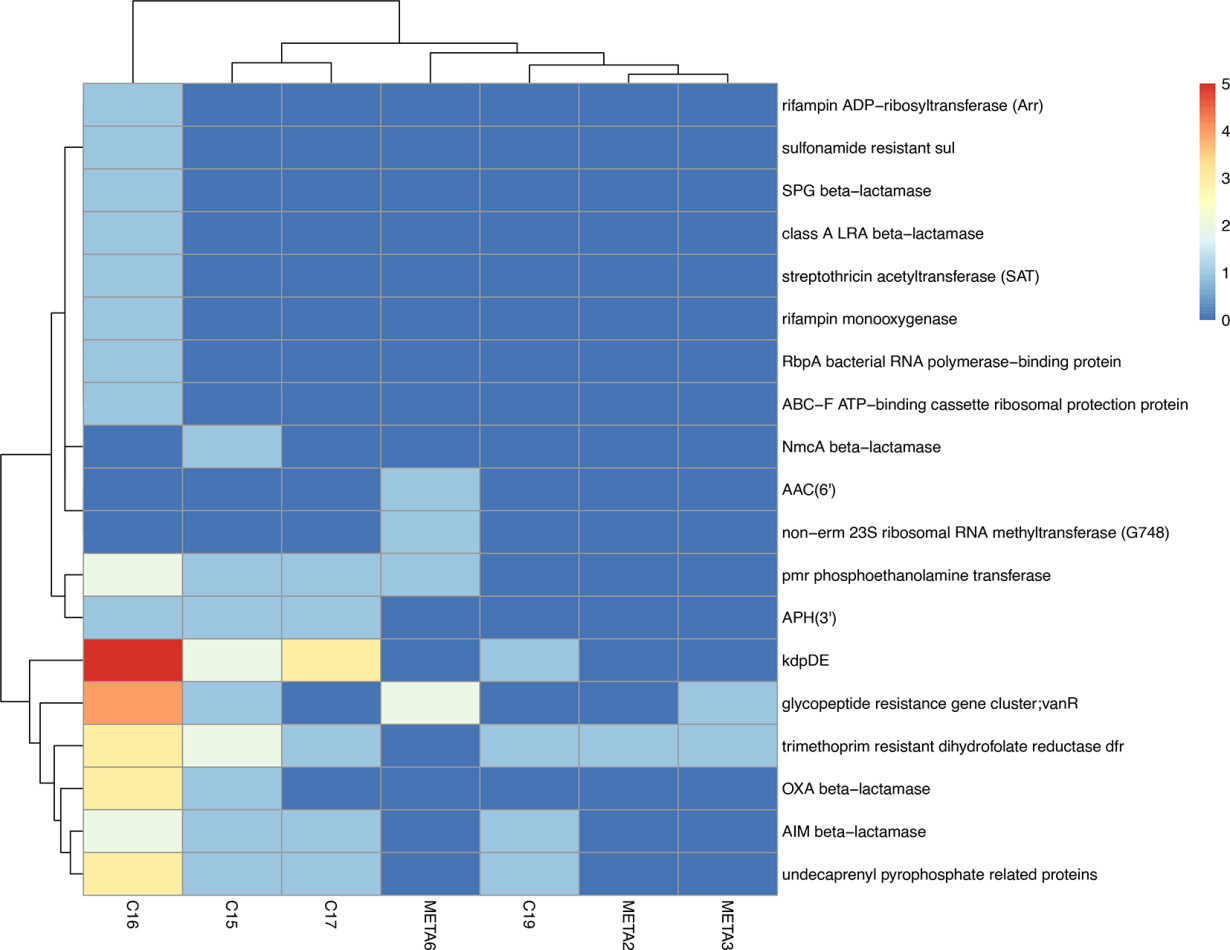
Heatmap of antibiotic resistance genes present in the samples.

Using the contigs assembled for taxonomic determination, we searched the CARD database for the presence of known antibiotic resistance genes. When comparing the number of antibiotic resistance genes that could be detected, we observed some differences. In the five samples of the first group with a lower sequencing depth (samples 2, 3, 6, 7, 8) between zero and nine resistance genes were detected per sample. In the four samples of the second group with a higher sequencing depth (samples 15, 16, 17, 19), between 14 and 115 resistance genes were detected (Fig. 3). A lower sequencing depth should lead to a decrease in the number of detected resistance genes, and low-abundance resistance genes might not be detected. This might explain why fewer resistance genes were detected in the biopsy samples sequenced with a lower sequencing depth [49].

We detected genes conferring resistance against trimethoprim, different aminoglycoside resistance genes (APH, AAC), vancomycin resistance gene clusters, members of different beta-lactamase families (OXA, NmcA, AIM, LRA, SPG), sulfonamide resistance genes, genes encoding enzymes changing cell wall permeability (Fig. 3) and, worryingly, an impressive amount of predicted antibiotic efflux pumps, belonging to the RND, MFS, SMR and ABC transporter families [55,57]. Some OXA beta-lactamase family members confer resistance predominantly to ceftazidime and additionally we detected genes conferring resistance to vancomycin, which is used together with ceftazidime in the treatment of endophthalmitis. Given the importance of the putative vancomycin and ceftazidime resistance genes for the treatment of endophthalmitis patients, we investigated them more closely. In three of the samples, we identified putative vancomycin resistance genes with about 40 % identity at the amino acid level and a coverage of 100 % and in one sample we detected vancomycin resistance genes with an identity of more than 90 % at the amino acid level and a coverage of 100 %. Genes encoding putative OXA beta-lactamases were detected in two of the samples, in both cases with identities of more than 90 % at the amino acid level and a coverage of 100 % to known resistance genes.

It is much more difficult to predict the specificity of the efflux pumps. Efflux pumps frequently have a broad substrate specificity and usually it is not possible to predict what the substrates are, although recently important advances have been made in this area [58]. In addition, it has been observed that mutations in the corresponding genes can modify the substrate specificities of the efflux pumps [50,59, 60]. Living organisms express a large variety of efflux pumps with broad specificity to deal with a large diversity of xenobiotics in the environment. These transporters, often with overlapping substrate specificities [61], recognize a wide range of substrates that may differ in structure, size or electrical charge and actively remove them from cells, thereby providing an essential survival strategy for the organism [62].

## Conclusions

In the present study, we sequenced total DNA extracted from biopsies obtained from eyes of acute endophthalmitis patients. In none of the samples was a single bacterial species dominant, but in all cases, we detected complex metagenomes, an observation which complicates the prediction of the agents causing the infection and the degradation of the tissue. Within this complexity we observed bacterial species that have been described as part of the skin microbiome and part of the ocular surface microbiome.

Using shotgun metagenomics, several resistance genes against a wide range of antibiotics were detected. Among these were resistance genes encoding OXA beta-lactamases which might confer resistance to ceftazidime and genes conferring resistance to vancomycin, two of the antibiotics currently used against endophthalmitis. It is also worrying that many genes encoding efflux pumps were identified.

Clinical metagenomics as a tool in ophthalmology has a large potential to reveal the variety of bacteria, viruses and fungi that can be present in endophthalmitis, but the analysis is still too time-consuming to be used as a routine tool in the clinic. It is probably of value to determine the metagenomes of thousands of patients worldwide to get a complete image of the organisms that can be present in endophthalmitis.

## Abbreviations

ABC: ATP-binding cassette
BCVA: best-corrected visual acuity
CARD: comprehensive antibiotic resistance database
DNA: deoxyribonucleic acid
EVS: endophthalmitis vitrectomy study
IVI: intravitreal injection
MFS: major facilitator superfamily
MIC: minimum inhibitory concentration
MXF: moxifloxacin
NCBI: National Center for Biotechnology Information
NGS: next-generation sequencing
PCR: polymerase chain reaction
PI: povidone iodine
RND: resistance-nodulation-division
rRNA: ribosomal ribonucleic acid
SMR: small multidrug resistance
VFDB: virulence factor database

## References

[1] Bowen RC, Zhou AX, Bondalapati S, Lawyer TW, Snow KB (2018). Comparative analysis of the safety and efficacy of intracameral cefuroxime, moxifloxacin and vancomycin at the end of cataract surgery: a meta-analysis. Br J Ophthalmol.

[2] Kernt M, Kampik A (2010). Endophthalmitis: pathogenesis, clinical presentation, management, and perspectives. Clin Ophthalmol.

[3] Nakashizuka H, Shimada H, Hattori T, Tanaka K, Kitagawa Y (2019). Intravitreal injection of 1.25% povidone iodine followed by vitrectomy using 0.025% povidone iodine irrigation for treating endophthalmitis. Transl Vis Sci Technol.

[4] Pathengay A, Khera M, Das T, Sharma S, Miller D (2012). Acute postoperative endophthalmitis following cataract surgery: a review. Asia Pac J Ophthalmol.

[5] Das T (2020). Endophthalmitis management: stain-culture, empirical treatment, and beyond. Asia Pac J Ophthalmol.

[6] Grzybowski A, Turczynowska M, Kuhn F (2018). The treatment of postoperative endophthalmitis: should we still follow the endophthalmitis vitrectomy study more than two decades after its publication?. Acta Ophthalmol.

[7] Gao H, Pennesi ME, Qiao X, Iyer MN, Wu SM (2006). Intravitreal moxifloxacin: retinal safety study with electroretinography and histopathology in animal models. Invest Ophthalmol Vis Sci.

[8] Kernt M, Neubauer AS, Ulbig MW, Kampik A, Welge-Lüssen U (2008). In vitro safety of intravitreal moxifloxacin for endophthalmitis treatment. J Cataract Refract Surg.

[9] Borroni D, Romano V, Kaye SB, Somerville T, Napoli L (2019). Metagenomics in ophthalmology: current findings and future prospectives. BMJ Open Ophthalmol.

[10] Gentile RC, Shukla S, Shah M, Ritterband DC, Engelbert M (2014). Microbiological spectrum and antibiotic sensitivity in endophthalmitis: a 25-year review. Ophthalmology.

[11] Hong BK, Lee CS, Van Gelder RN, Garg SJ (2015). Emerging techniques for pathogen discovery in endophthalmitis. Curr Opin Ophthalmol.

[12] Bispo PJM, de Melo GB, Hofling-Lima AL, Pignatari ACC (2011). Detection and gram discrimination of bacterial pathogens from aqueous and vitreous humor using real-time PCR assays. Invest Ophthalmol Vis Sci.

[13] Joseph CR, Lalitha P, Sivaraman KR, Ramasamy K, Behera UC (2012). Real-time polymerase chain reaction in the diagnosis of acute postoperative endophthalmitis. Am J Ophthalmol.

[14] Sugita S, Shimizu N, Watanabe K, Katayama M, Horie S (2011). Diagnosis of bacterial endophthalmitis by broad-range quantitative PCR. Br J Ophthalmol.

[15] Mishra D, Satpathy G, Chawla R, Paliwal D, Panda SK (2021). Targeted metagenomics using next generation sequencing in laboratory diagnosis of culture negative endophthalmitis. Heliyon.

[16] Gallon P, Parekh M, Ferrari S, Fasolo A, Ponzin D (2019). Metagenomics in ophthalmology: Hypothesis or real prospective?. Biotechnol Rep (Amst).

[17] Bachmann NL, Rockett RJ, Timms VJ, Sintchenko V (2018). Advances in clinical sample preparation for identification and characterization of bacterial pathogens using metagenomics. Front Public Health.

[18] Chiu CY, Miller SA (2019). Clinical metagenomics. Nat Rev Genet.

[19] Crofts TS, Gasparrini AJ, Dantas G (2017). Next-generation approaches to understand and combat the antibiotic resistome. Nat Rev Microbiol.

[20] Mullany P (2014). Functional metagenomics for the investigation of antibiotic resistance. Virulence.

[21] Zhu J, Xia H, Tang R, Ng TK, Yao F (2022). Metagenomic next-generation sequencing detects pathogens in endophthalmitis patients. Retina.

[22] Jabs DA, Nussenblatt RB, Rosenbaum JT, Standardization of Uveitis Nomenclature (SUN) Working Group (2005). Standardization of uveitis nomenclature for reporting clinical data. Results of the First International Workshop. Am J Ophthalmol.

[23] Aydin E, Kazi AA, Peyman GA, Esfahani MR (2006). Intravitreal toxicity of moxifloxacin. Retina.

[24] Jacobs DJ, Grube TJ, Flynn HW, Greven CM, Pathengay A (2013). Intravitreal moxifloxacin in the management of Ochrobactrum intermedium endophthalmitis due to metallic intraocular foreign body. Clin Ophthalmol.

[25] Wood DE, Lu J, Langmead B (2019). Improved metagenomic analysis with Kraken 2. Genome Biol.

[26] Langmead B, Salzberg SL (2012). Fast gapped-read alignment with Bowtie 2. Nat Methods.

[27] Prjibelski A, Antipov D, Meleshko D, Lapidus A, Korobeynikov A (2020). Using SPAdes de novo assembler. Curr Protoc Bioinform.

[28] Seemann T (2014). Prokka: rapid prokaryotic genome annotation. Bioinformatics.

[29] Breitwieser FP, Salzberg SL (2020). Pavian: interactive analysis of metagenomics data for microbiome studies and pathogen identification. Bioinformatics.

[30] Altschul SF, Gish W, Miller W, Myers EW, Lipman DJ (1990). Basic local alignment search tool. J Mol Biol.

[31] Chen L, Zheng D, Liu B, Yang J, Jin Q (2016). VFDB 2016: hierarchical and refined dataset for big data analysis--10 years on. Nucleic Acids Res.

[32] Alcock BP, Raphenya AR, Lau TTY, Tsang KK, Bouchard M (2020). CARD 2020: antibiotic resistome surveillance with the comprehensive antibiotic resistance database. Nucleic Acids Res.

[33] Sui H-Y, Weil AA, Nuwagira E, Qadri F, Ryan ET (2020). Impact of DNA extraction method on variation in human and built environment microbial community and functional profiles assessed by shotgun metagenomics sequencing. Front Microbiol.

[34] Ma L, Jakobiec FA, Dryja TP (2019). A review of next-generation sequencing (NGS): applications to the diagnosis of ocular infectious diseases. Semin Ophthalmol.

[35] Dong Q, Brulc JM, Iovieno A, Bates B, Garoutte A (2011). Diversity of bacteria at healthy human conjunctiva. Invest Ophthalmol Vis Sci.

[36] Ormeño-Orrillo E, Martínez-Romero E (2019). A genomotaxonomy view of the *Bradyrhizobium* genus. Front Microbiol.

[37] Anand AR, Therese KL, Madhavan HN (2000). Spectrum of aetiological agents of postoperative endophthalmitis and antibiotic susceptibility of bacterial isolates. Indian J Ophthalmol.

[38] Barry P, Seal DV, Gettinby G, Lees F, Peterson M (2006). ESCRS study of prophylaxis of postoperative endophthalmitis after cataract surgery: preliminary report of principal results from a European multicenter study. J Cataract Refract Surg.

[39] Dave VP, Pathengay A, Nishant K, Pappuru RR, Sharma S (2017). Clinical presentations, risk factors and outcomes of ceftazidime-resistant Gram-negative endophthalmitis. Clin Exp Ophthalmol.

[40] Han DP, Vine AK, Blodi BA, Elner SG, Johnson MW (1996). Microbiologic factors and visual outcome in the Endophthalmitis Vitrectomy study. Am J Ophthalmol.

[41] Kunimoto DY, Das T, Sharma S, Jalali S, Majji AB (1999). Microbiologic spectrum and susceptibility of isolates: part I. Postoperative endophthalmitis. Endophthalmitis Research Group. Am J Ophthalmol.

[42] Sheng Y, Sun W, Gu Y, Lou J, Liu W (2011). Endophthalmitis after cataract surgery in China, 1995-2009. J Cataract Refract Surg.

[43] Wong TY, Chee SP (2004). The epidemiology of acute endophthalmitis after cataract surgery in an Asian population. Ophthalmology.

[44] Lee AY, Akileswaran L, Tibbetts MD, Garg SJ, Van Gelder RN (2015). Identification of torque teno virus in culture-negative endophthalmitis by representational deep DNA sequencing. Ophthalmology.

[45] Silva DCM, Moreira-Silva E dos S, Gomes J de A, Fonseca F da, Correa-Oliveira R (2010). Clinical signs, diagnosis, and case reports of Vaccinia virus infections. Braz J Infect Dis.

[46] Goolam Mahomed T, Peters RPH, Allam M, Ismail A, Mtshali S (2021). Lung microbiome of stable and exacerbated COPD patients in Tshwane, South Africa. Sci Rep.

[47] Deshmukh D, Joseph J, Chakrabarti M, Sharma S, Jayasudha R (2019). New insights into culture negative endophthalmitis by unbiased next generation sequencing. Sci Rep.

[48] Durand ML (2013). Endophthalmitis. Clin Microbiol Infect.

[49] Pereira-Marques J, Hout A, Ferreira RM, Weber M, Pinto-Ribeiro I (2019). Impact of host DNA and sequencing depth on the taxonomic resolution of whole metagenome sequencing for microbiome analysis. Front Microbiol.

[50] Nikaido H (1998). Multiple antibiotic resistance and efflux. Curr Opin Microbiol.

[51] Ali J, Johansen W, Ahmad R (2024). Short turnaround time of seven to nine hours from sample collection until informed decision for sepsis treatment using nanopore sequencing. Sci Rep.

[52] Hu Y, Yang X, Qin J, Lu N, Cheng G (2013). Metagenome-wide analysis of antibiotic resistance genes in a large cohort of human gut microbiota. Nat Commun.

[53] van Schaik W (2015). The human gut resistome. Philos Trans R Soc Lond B Biol Sci.

[54] Sommer MOA, Munck C, Toft-Kehler RV, Andersson DI (2017). Prediction of antibiotic resistance: time for a new preclinical paradigm?. Nat Rev Microbiol.

[55] Larsson DGJ, Flach CF (2022). Antibiotic resistance in the environment. Nat Rev Microbiol.

[56] Zhang Z, Zhang Q, Wang T, Xu N, Lu T (2022). Assessment of global health risk of antibiotic resistance genes. Nat Commun.

[57] Urban-Chmiel R, Marek A, Stępień-Pyśniak D, Wieczorek K, Dec M (2022). Antibiotic resistance in bacteria-a review. Antibiotics.

[58] Zwama M, Nishino K (2021). Ever-adapting RND efflux pumps in gram-negative multidrug-resistant pathogens: a race against time. Antibiotics.

[59] Gottesman MM, Fojo T, Bates SE (2002). Multidrug resistance in cancer: role of ATP-dependent transporters. Nat Rev Cancer.

[60] Gottesman MM, Ling V (2006). The molecular basis of multidrug resistance in cancer: the early years of P-glycoprotein research. FEBS Lett.

[61] Zwama M, Yamaguchi A (2018). Molecular mechanisms of AcrB-mediated multidrug export. Res Microbiol.

[62] Tal N, Schuldiner S (2009). A coordinated network of transporters with overlapping specificities provides a robust survival strategy. Proc Natl Acad Sci U S A.

